# Crystal structures of a novel NNN pincer ligand and its dinuclear titanium(IV) alkoxide pincer complex

**DOI:** 10.1107/S2056989016019964

**Published:** 2017-01-06

**Authors:** Jakub Pedziwiatr, Ion Ghiviriga, Khalil A. Abboud, Adam S. Veige

**Affiliations:** a Department of Chemistry, Center for Catalysis, University of Florida, Gainesville, FL 32611, USA

**Keywords:** crystal structure, pincer ligand, tridentate ligands, dinuclear titanium complex, organometallics

## Abstract

This report describes a synthetic protocols and the crystal structures involving a novel pincer-type H_3_[NNN] ligand, namely di-μ-bromido-μ-{2-(2,2-di­methylpropanimido­yl)-*N*-[2-(2,2-di­methyl­propanimido­yl)-4-methyl­phen­yl]-4-methylaniline}-bis­[(diethyl ether)lithium], [Li_2_Br_2_(C_24_H_33_N_3_)(C_4_H_10_O)_2_] (**1**) and a dinuclear metal complex, namely di-μ-bromido-2:3κ^4^ *Br*:*Br*-bis­{2-(2,2-di­methylpropanimido­yl)-*N*-[2-(2,2-di­methyl­propanimido­yl)-4-methyl­phen­yl]-4-methylaniline}-1κ^3^ *N*,*N*′,*N*′′;4κ^3^ *N*,*N*′,*N*′′-tetra-μ-iso­propano­lato-1:2κ^4^ *O*:*O*;3:4κ^4^ *O*:*O*-diiso­propano­lato-1κ*O*,4κ*O*-2,3-dilithium-1,4-dititanium, [Li_2_Ti_2_Br_2_(C_24_H_32_N_3_)_2_(C_3_H_7_O)_6_] or {[NHNNH]Ti(O^*i*^Pr)_3_(LiBr)_2_}_2_ (**2**). Complex **1**, which sits on a twofold rotation axis, is a rare example of a pincer-type ligand which bears ketimine side arms. A unique feature of complex **1** is that the ketimine N atoms have an LiBr(Et_2_O) fragment bonded to them, with the Li atom adopting a distorted tetra­hedral geometry. This particular fragment creates an LiBr bridge between the two ketimine sidearms, which leads to a cage-type appearance of the ligand. Complex **2** consists of the previously described ligand and a Ti^IV^ metal atom in an octa­hedral environment, and is located on an inversion center. Complex **2** crystallizes as a dinuclear species with the metal atoms being bridged by an LiBr entity [the Br atoms are disordered and refined in two positions with their site occupation factors refining to 0.674 (12)/0.372 (12)], and the Li cation being bonded to the isopropoxide O atoms (Li having a tetra­hedral coordination as in **1**). The organic ligand of compound **2** exhibits disorder in its periphery groups; isopropyl and *tert*-butyl groups (occupation factors fixed at 0.6/0.4). The novel [NNN]H_3_ pincer-type ligand was characterized by multinuclear and multidimensional NMR, HRMS and X-ray crystallography. The dinuclear metal complex **2** was characterized by X-ray crystallography. Although each structure exhibits donor N—H groups, no hydrogen bonding is found in either one, perhaps due to the bulky groups around them. One of the ethyl groups of the ether ligand of **1** is disordered and refined in two parts with site-occupation factors of 0.812 (8) and 0.188 (8). One and a half toluene solvent mol­ecules are also present in the asymmetric unit of **2**. The toluene mol­ecules were significantly disordered and could not be modeled properly, thus *SQUEEZE* [Spek (2015 ▸). *Acta Cryst.* C**71**, 9–18] was used to remove their contributions to the overall intensity data.

## Abstract 2

The title compound is an LiBr-bridged Ti^IV^ alkoxide dimer, supported by a novel monoanionic [NNN] pincer-type ligand. The bis­[2-(1-imino-2,2-di­methyl­prop­yl)-4-methyl­phen­yl]amine ligand is the first reported ligand that bears hydrogen atoms on its ketimine side arms.

## Chemical context   

Pincer ligands occupy the meridional coordination sites on a metal ion and were first introduced by Moulton and Shaw in 1976. In the original system, the pincer ligand 2,6-*bis*[(*di-t*-butyl­phosphino)meth­yl]phenyl binds to the late transition metals Ni, Pd, Pt, Rh, and Ir through the deprotonated aromatic carbon and the pendant –P*R*_2_ side arms (*R* = *t-*but­yl) (Moulton & Shaw, 1976 ▸). Under the HSAB theory, this particular arrangement can be viewed as a soft–hard–soft coordination mode. Since this discovery, the library of tridentate ligands that exhibit this unique meridional coord­in­ation of a metal atom has been extended not only by additional monoanionic pincer and pincer-type ligands, but as well by numerous neutral, dianionic and trianionic pincer-type ligands (Van Koten, 2013 ▸; Gunanathan & Milstein, 2011 ▸; O’Reilly & Veige, 2014 ▸). Recent advances in the chemistry of metal complexes supported by trianionic pincer and pincer-type ligands which exhibit a unique hard–hard–hard binding mode (Sarkar *et al.*, 2008 ▸) highlight their potential for applications as catalysts in polymerizations (McGowan *et al.*, 2013 ▸), alkene isomerizations (McGowan *et al.*, 2011 ▸), and as catalytic group or atom-transfer reagents (O’Reilly *et al.*, 2009 ▸).
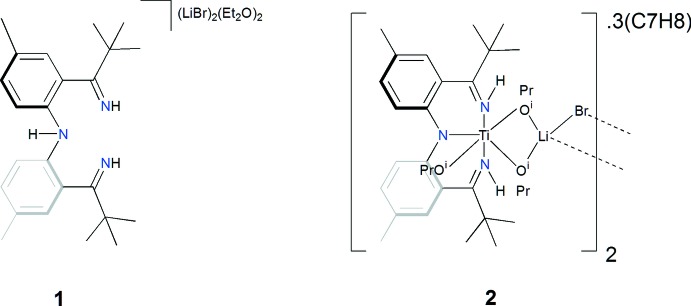


Monoanionic di­aryl­amino [NNN] ligands with imine functionality on the flanking side arms were reported in 1978 by Black and Rothnie (Black & Rothnie, 1978 ▸), and are gaining inter­est as evidenced by newly introduced systems in recent years. In the present work, we introduce a protocol for the synthesis and characterization of a novel [NNN]H_3_ pincer-type ligand that involves the addition of a nitrile to an ar­yl–lithium salt, a protocol described by Parham and coworkers (Parham *et al.*, 1978 ▸).

## Structural commentary   

**Figure 1:**
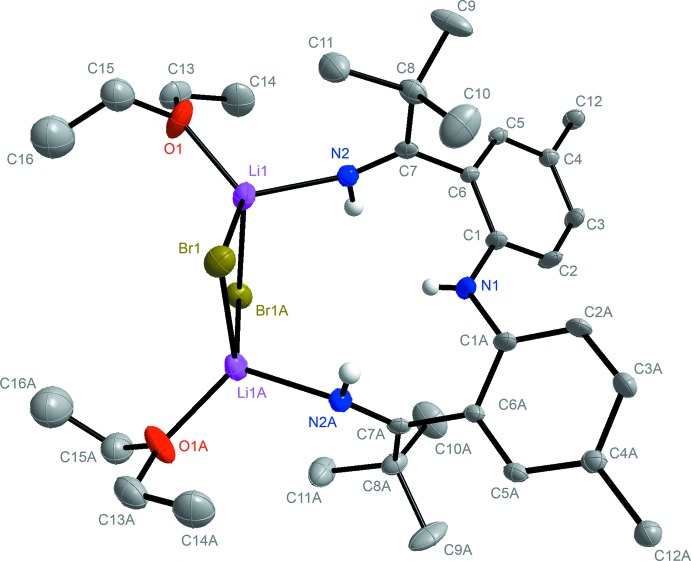
The mol­ecular structure of [NNN]H_3_ (**1**), with C-bound H atoms and minor components of disorder removed for clarity. Symmetry code: (A) −*x* + 1, *y*, −*z* + 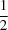.

Ketimine ligands typically possess a bulky group (such as ^*t*^Bu) on their N atoms. The ligand moiety of **1** (Fig. 1 ▸) is unique in that it contains a proton in the terminal position. The complete molecule of **1** is located about a twofold rotation axis. The coordinated Li atoms exhibit an N2—Li2 bond length of 2.065 (7) Å and a N3—Li1 bond length of 2.065 (7) Å. The two lithium ions are both bridged by two bromides with an Li1—Br1 bond length of 2.504 (6) Å and an Li—Br1(−*x* + 1, *y*, −*z* + 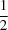) bond length of 2.531 (7) Å. Furthermore, both coordinated lithium ions carry a bound Et_2_O solvent mol­ecule, each with an Li—O bond length of 1.961 (7) Å. The short C=N bond length of 1.277 (4) Å is comparable to reported C=N bond lengths. For instance the C=N bond length in furazan is 1.29 Å (Allen *et al.*, 1987 ▸).

Similar to its solid-state structure, **1** exhibits *C*_2_ symmetry in solution. The ^1^H NMR spectrum in CDCl_3_ (see Supporting information) exhibits a singlet at 2.26 ppm attributable to the methyl groups on the aryl backbone of the ligand framework. Another characteristic singlet that appears at 1.20 ppm has three times the intensity of the backbone CH_3_ and is attrib­utable to the *tert*-butyl CH_3_ protons residing on each ligand arm. Furthermore, the ^1^H NMR spectrum exhibits a quartet at 3.48 ppm and a triplet at 1.20 ppm, both signals can be assigned to the –C*H_2_* and –C*H_3_* groups of bound Et_2_O. The central backbone N—H resonates as a singlet at 5.32 ppm, and the ketimine N—H protons resonate at 9.42 ppm. ^1^H–^15^N *g*HMBC indirect detection demonstrates that the central nitro­gen resonates at 77.00 ppm. In contrast, the chemical shifts of the ketimine nitro­gen atoms are not observable. Furthermore, in a NOESY1D experiment the *tert*-butyl CH_3_ groups show an nOe with the Et_2_O –CH_3_ group when irradiated selectively at 1.28 ppm. From the occurrence of this nOe, it can be concluded that one Et_2_O mol­ecule is bonded to every lithium atom.

**Figure 2:**
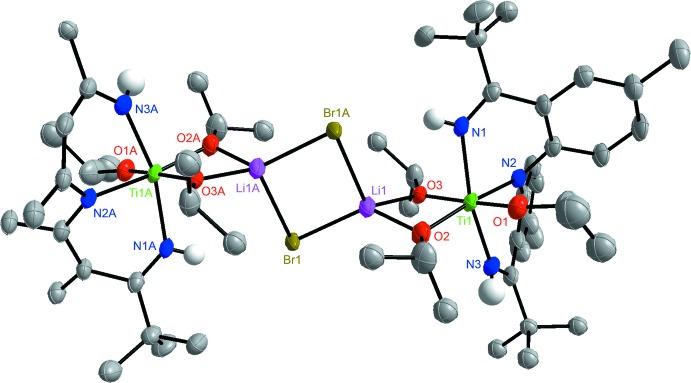
The mol­ecular structure of the dinuclear {[NHNNH]Ti(OiPr)_3_(LiBr)_2_}_2_ complex (**2**), with all hydrogen atoms bound to C atoms removed for clarity. Symmetry code: (A) −*x*, −*y* + 1, −*z* + 1.

In the solid state, complex **2** is located on an inversion center (Figs. 2 ▸ and 3 ▸) and the Ti^IV^ core exhibits a slightly distorted octa­hedral environment. The N2—Ti bond length of 2.069 (2) Å confirms that the central pincer nitro­gen atom is deprotonated. The slightly elongated N1—Ti and N3—Ti bonds of 2.136 (3) and 2.130 (3) Å are indicative of an L-type bonding of the ket-mine nitro­gen atoms. The bond lengths and the fact that the titanium metal atom is coordinated by three isopropoxide ligands supports the claim that the [NNN] ligand within **2** must be monoanionic with both ketimine N—H protons still present. The Ti—O1, Ti—O2 and Ti—O3 bond lengths are 1.805 (2), 1.901 (2) and 1.934 (2) Å, respectively. The increase in bond length between Ti—O2 and Ti—O3 in comparison to Ti—O1 is attributed to the coordination of Li to O2 and O3. While the O3—Ti—O1 bond angle of 173.58 (9)° deviates slightly from the optimal angle of 180°, the angle N1—Ti—N3 is 160.94 (11)°. This distortion is due to the short bond length that can be found in a C=N bond. The dinuclear complex also exhibits four disordered regions. The isopropyl groups on C25, C28, C31 and the *tert*-butyl group on C21 are all disordered and were refined in two parts. The bridging Br ligands are also disordered and were refined in two parts; namely Br1 and Br2.

## Experimental   

Unless specified otherwise, all manipulations were performed under an inert atmosphere using standard Schlenk or glovebox techniques. Glassware was pre-dried in an oven before use. Pentane, toluene, and diethyl ether (Et_2_O) were dried using a GlassContours drying column. Chloro­from-*d*_1_ (Cambridge Isotopes) was dried over anhydrous CaCl_2_; vacuum transferred, passed over a plug of basic alumina, and stored over 4 Å mol­ecular sieves. Di-*p-*tolyl­amine, ^*n*^BuLi (2.5 *M* in hexa­nes), titanium(IV)isopropoxide, and HCl (1 *M* in Et_2_O) were purchased from Sigma Aldrich and used as received. Tri­methyl­aceto­nitrile was vacuum distilled and freeze pump thawed prior to use. Bis(2-bromo-4-methyl­phen­yl)amine was prepared by literature methods (Corey *et al.*, 2010 ▸).

### Synthesis and crystallization of title compound 1   

In a nitro­gen-filled glove-box, a glass vial was charged with bis­(2-bromo-4-methyl­phen­yl)amine (0.125 g, 0.35 mmol), 3.0 mL of Et_2_O. 3.1 eq. ^*n*^BuLi (2.5 *M* in hexa­nes) (0.44 mL, 1.1 mmol) was added dropwise to a stirring solution of bis­(2-bromo-4-methyl­phen­yl)amine. The reaction mixture color changed from colorless to yellow. After stirring for 120 min, pivalo­nitrile was added dropwise, resulting in a color change from yellow to orange. After an additional 180 min of stirring, excess HCl (1 *M* in Et_2_O) was added dropwise, resulting in a color change from orange to yellow and the formation of a white microcrystalline powder. The pale-yellow solution was filtered through Celite™. The volatiles in the resulting filtrate were removed *in vacuo* and the oily residue was triturated three times (3 × 2 mL) with pentane. Single crystals were obtained by cooling a concentrated toluene solution of **1** to 238 K. Yield: 0.091 g (0.14 mmol, 41%). ^1^H NMR (500 MHz, CDCl_3_, 298 K): *δ* = 1.20 (*t*, 12H, *CH_3_*(Et_2_O)_2_), 1.28 (*s*, 18H, *CH_3_*(C_4_H_9_)_2_), 2.26 (*s*, 6H, –*CH_3_*), 3.48 [*q*, 8H, *CH_2_*(Et_2_O)_2_], 6.79 (*s*, 2H, Ar-*H*), 6.96 (*dd*, 4H, Ar-*H*,) ppm. ^1^H–^13^C gHMBC NMR (500 MHz, CDCl_3_, 298 K): δ(ppm) = 15.3 [*s*, CH_3_(Et_2_O) C], 20.2 (*s*, CH_3_), 28.1 [*s*, *CH_3_*(C_4_H_9_)], 40.9 [*s*, –*C–*(C_4_H_9_)], 65.8 [*s*, CH_2_(Et_2_O) C], 119.8 (*s*, Ar C), 127.6 (*s*, Ar C), 129.5 (*s*, Ar C), 129.7 (*s*, Ar C), 132.3 (*s*, Ar C), 137.3 (*s*, Ar C) and 190.6 (*s*, N=C); HRMS calculated (found) for C_24_H_33_N_3_ (*M* ^+^) 364.2747 (364.2755).

### Synthesis and crystallization of title compound 2   

In a nitro­gen-filled glove-box, a glass vial was charged with [NNN]H_3_(LiBr)_2_ (**1**) (0.075 g, 0.118 mmol), and 3 mL of Et_2_O. 1.1 eq. Ti(O^*i*^Pr)_4_ (38.5 µL, 0.130 mmol) was added dropwise to a stirring solution of **1**. The reaction mixture changed color instantaneously from yellow to dark red. After stirring for 120 min, the dark-red solution was filtered through Celite™. The volatiles in the resulting filtrate were removed *in vacuo* and the resulting oily residue was washed three times (3 × 2 mL) with pentane. Single crystals were obtained by preparing a concentrated solution of the oily complex **2** in toluene and cooling it for two weeks at 238 K. Yield: 0.107 g (0.081 mmol, 69%).

## Refinement details complex 1   

Crystal data, data collection and structure refinement details are summarized in Table 1 ▸. The non-H atoms were refined with anisotropic displacement parameters and all of the H atoms were calculated in idealized positions (C—H = 0.93/1.00 Å) and refined riding on their parent atoms with *U*_iso_(H)= 1.2/1.5*U*_eq_(C), except for the –N—H hydrogen atoms which were obtained from a difference Fourier map and refined freely. The dimer complex is located on a twofold rotation axis of symmetry and thus only a half is contained in the asymmetric unit. One ethyl group of the Li-coordinating ether ligand is disordered and was refined in two parts (C15–C16/C15′–C16′). Their site-occupation factors dependently refined to 0.812 (8) and 0.188 (8), for the major and minor parts, respectively.

### Refinement details complex 2   

The non-H atoms were refined with anisotropic displacement parameters and all of the H atoms were calculated in idealized positions (C—H = 0.93/1.00 Å) and refined riding on their parent atoms with *U*_iso_(H)= 1.2/1.5*U*_eq_(C), except for the –N—H hydrogen atoms which were obtained from a difference-Fourier map and refined freely. The Ti dimer is located on an inversion center and thus a half dimer is present in the asymmetric unit. One and a half toluene solvent mol­ecules are also present in the asymmetric unit. The half toluene mol­ecule is disordered around inversion symmetry while the one in a general position is disordered in two parts. The toluene mol­ecules were significantly disordered and could not be modeled properly, thus *SQUEEZE* (Spek, 2015 ▸), a part of the *PLATON* (Spek, 2009 ▸) package of crystallographic software, was used to calculate the solvents’ disorder areas and remove their contributions to the overall intensity data. The disordered solvents area is centered around the 0.0, 0.0, 0.0 position and showing an estimated total of 151 electrons and a void volume of 586 Å^3^. The dimer also exhibits four disordered regions. The isopropyl groups on C25, C28, C31 and the *t*-butyl group on C21 are all disordered and were refined in two parts with their site occupation factors fixed to 0.6/0.4 in the final refinement model. The bridging Br ligands are also disordered and refined in two parts, Br1 and Br2, to values of 0.674 (12) and 0.326 (12), respectively. The –N—H hydrogen atoms were obtained from a difference-Fourier map and refined freely.

## Supplementary Material

Crystal structure: contains datablock(s) 1, 2. DOI: 10.1107/S2056989016019964/lh5823sup1.cif

Structure factors: contains datablock(s) 1. DOI: 10.1107/S2056989016019964/lh58231sup2.hkl

Structure factors: contains datablock(s) 2. DOI: 10.1107/S2056989016019964/lh58232sup3.hkl

Supporting information file. DOI: 10.1107/S2056989016019964/lh5823sup4.pdf

CCDC references: 1523135, 1523134

Additional supporting information:  crystallographic information; 3D view; checkCIF report

## Figures and Tables

**Figure 3:**
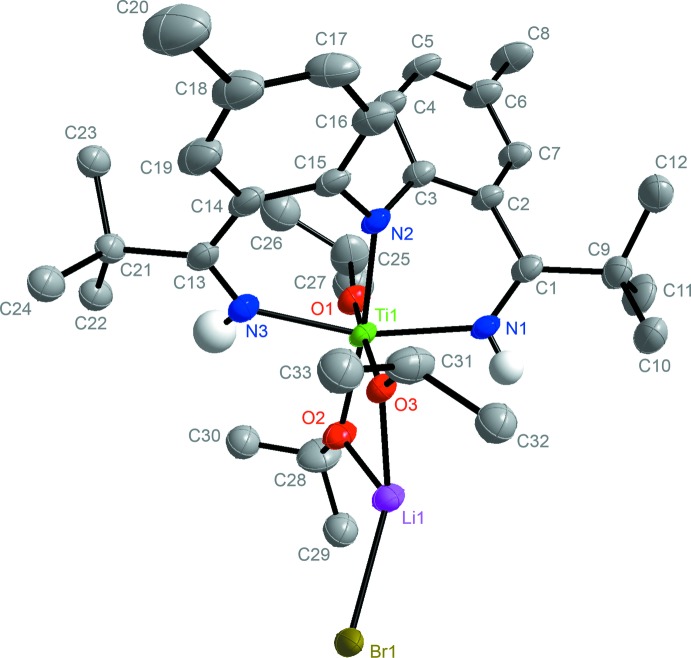
The mol­ecular structure of one half of the {[NHNNH]Ti(OiPr)_3_(LiBr)_2_}_2_ dimer (**2**)with hydrogen atoms removed for clarity.

**Table 1:** Experimental details

	**1**	**2**
Crystal data		
Chemical formula	[Li_2_Br_2_(C_24_H_33_N_3_)(C_4_H_10_O)_2_]	[Li_2_Ti_2_Br_2_(C_24_H_32_N_3_)_2_(C_3_H_7_O)_6_]·1.5C_7_H_8_
*M*_r_	685.47	1761.71
Crystal system, space group	Orthorhombic, *P* *b* *c* *n*	Triclinic, *P* 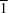
Temperature (K)	100	100
*a*, *b*, *c* (Å)	11.8301 (7), 22.3946 (13), 13.4254 (8)	12.2546 (3), 12.7240 (3), 15.9477 (5)
α, β, γ (°)	90, 90, 90	75.8613 (15), 68.0449 (15), 83.2200 (17)
*V* (Å^3^)	3556.8 (4)	2235.49 (11)
*Z*	4	1
Radiation type	Mo *K*α	Cu *K*α
μ (mm^−1^)	2.31	3.00
Crystal size (mm)	0.15 × 0.13 × 0.06	0.25 × 0.20 × 0.04

Data collection		
Diffractometer	Bruker APEXII DUO	Bruker APEXII DUO
Absorption correction	Analytical [based on measured indexed crystal faces (*SHELXTL*; Sheldrick, 2008 ▸)]	Analytical [based on measured indexed crystal faces (*SHELXTL*; Sheldrick, 2008 ▸)]
*T*_min_, *T*_max_	0.758, 0.891	0.667, 0.890
No. of measured, independent and observed [*I* > 2σ(*I*)] reflections	45357, 4096, 3116	30419, 7594, 6139
*R*_int_	0.050	0.083
(sin θ/λ)_max_ (Å^−1^)	0.650	0.595

Refinement		
*R*[*F* ^2^ > 2σ(*F* ^2^)], *wR*(*F* ^2^), *S*	0.052, 0.140, 1.03	0.058, 0.172, 1.10
No. of reflections	4096	7594
No. of parameters	189	386
No. of restraints	3	324
H-atom treatment	H atoms treated by a mixture of independent and constrained refinement	H atoms treated by a mixture of independent and constrained refinement
Δρ_max_, Δρ_min_ (e Å^−3^)	1.01, −0.79	0.72, −0.43

Computer programs: *APEX2* and *SAINT* (Bruker, 2008 ▸), *SHELXT2013* (Sheldrick, 2015*a* ▸), *SHELXTL2014* (Sheldrick, 2008 ▸), *SHELXL2013* (Sheldrick, 2015*b* ▸) and *DIAMOND* (Brandenburg, 2014 ▸).

## supplementary crystallographic information

### (1) Di-µ-bromido-µ-{2-(2,2-dimethylpropanimidoyl)-N-[2-(2,2-dimethylpropanimidoyl)-4-methylphenyl]-4-methylaniline}-bis[(diethyl ether)lithium] Crystal data

**Table:**

[Li_2_Br_2_(C_24_H_33_N_3_)(C_4_H_10_O)_2_]	*D*_x_ = 1.280 Mg m^−^^3^
*M**_r_* = 685.47	Mo *K*α radiation, λ = 0.71073 Å
Orthorhombic, *P**b**c**n*	Cell parameters from 9991 reflections
*a* = 11.8301 (7) Å	θ = 2.0–28.0°
*b* = 22.3946 (13) Å	µ = 2.31 mm^−^^1^
*c* = 13.4254 (8) Å	*T* = 100 K
*V* = 3556.8 (4) Å^3^	Platelets, orange
*Z* = 4	0.15 × 0.13 × 0.06 mm
*F*(000) = 1432	

### (1) Di-µ-bromido-µ-{2-(2,2-dimethylpropanimidoyl)-N-[2-(2,2-dimethylpropanimidoyl)-4-methylphenyl]-4-methylaniline}-bis[(diethyl ether)lithium] Data collection

**Table:**

Bruker APEXII DUO diffractometer	3116 reflections with *I* > 2σ(*I*)
Radiation source: fine-focus sealed tube	*R*_int_ = 0.050
phi and ω scans	θ_max_ = 27.5°, θ_min_ = 1.8°
Absorption correction: analytical [based on measured indexed crystal faces (SHELXTL; Sheldrick, 2008)]	*h* = −14→15
*T*_min_ = 0.758, *T*_max_ = 0.891	*k* = −29→28
45357 measured reflections	*l* = −17→17
4096 independent reflections	

### (1) Di-µ-bromido-µ-{2-(2,2-dimethylpropanimidoyl)-N-[2-(2,2-dimethylpropanimidoyl)-4-methylphenyl]-4-methylaniline}-bis[(diethyl ether)lithium] Refinement

**Table:**

Refinement on *F*^2^	Primary atom site location: structure-invariant direct methods
Least-squares matrix: full	Secondary atom site location: difference Fourier map
*R*[*F*^2^ > 2σ(*F*^2^)] = 0.052	Hydrogen site location: mixed
*wR*(*F*^2^) = 0.140	H atoms treated by a mixture of independent and constrained refinement
*S* = 1.03	*w* = 1/[σ^2^(*F*_o_^2^) + (0.0691*P*)^2^ + 7.3069*P*] where *P* = (*F*_o_^2^ + 2*F*_c_^2^)/3
4096 reflections	(Δ/σ)_max_ < 0.001
189 parameters	Δρ_max_ = 1.01 e Å^−^^3^
3 restraints	Δρ_min_ = −0.79 e Å^−^^3^

### (1) Di-µ-bromido-µ-{2-(2,2-dimethylpropanimidoyl)-N-[2-(2,2-dimethylpropanimidoyl)-4-methylphenyl]-4-methylaniline}-bis[(diethyl ether)lithium] Special details

**Table:**

Geometry. All esds (except the esd in the dihedral angle between two l.s. planes) are estimated using the full covariance matrix. The cell esds are taken into account individually in the estimation of esds in distances, angles and torsion angles; correlations between esds in cell parameters are only used when they are defined by crystal symmetry. An approximate (isotropic) treatment of cell esds is used for estimating esds involving l.s. planes.
Refinement. All H atoms were positioned geometrically ( C—H = 0.93/1.00 Å) and allowed to ride with *U*_iso_(H)= 1.2/1.5*U*_eq_(C). Methyl ones were allowed to rotate around the corresponding C—C. The dimer complex is located on a 2-fold rotation axis of symmetry thus only a half is contained in the asymmetric unit. One ethyl group of the Li coordinated ether ligand is disordered and was refined in two parts (C15-C16/C15'-C16'). Their site occupation factors dependently refined to 0.812 (8) and 0.188 (8) for the major and minor parts, respectively. The nitrogen protons were obtained from a Difference Fourier map and refined freely.

### (1) Di-µ-bromido-µ-{2-(2,2-dimethylpropanimidoyl)-N-[2-(2,2-dimethylpropanimidoyl)-4-methylphenyl]-4-methylaniline}-bis[(diethyl ether)lithium] Fractional atomic coordinates and isotropic or equivalent isotropic displacement parameters (Å^2^)

**Table:**

	*x*	*y*	*z*	*U*_iso_*/*U*_eq_	Occ. (<1)
Br1	0.61932 (3)	0.39333 (2)	0.34805 (3)	0.03136 (14)	
O1	0.6756 (3)	0.44324 (13)	0.0817 (2)	0.0480 (8)	
N1	0.5000	0.1968 (2)	0.2500	0.0340 (12)	
H1	0.5000	0.226 (2)	0.2500	0.014 (15)*	
Li1	0.5936 (6)	0.3850 (3)	0.1634 (4)	0.0299 (13)	
N2	0.6013 (3)	0.29847 (13)	0.1107 (2)	0.0236 (6)	
H2	0.529 (3)	0.2921 (16)	0.095 (3)	0.020 (9)*	
C1	0.5220 (3)	0.16765 (15)	0.1599 (2)	0.0273 (8)	
C2	0.4665 (4)	0.11449 (16)	0.1360 (3)	0.0358 (9)	
H2A	0.4154	0.0974	0.1826	0.043*	
C3	0.4844 (3)	0.08642 (16)	0.0463 (3)	0.0317 (8)	
H3A	0.4463	0.0500	0.0326	0.038*	
C4	0.5571 (3)	0.11027 (14)	−0.0246 (2)	0.0223 (7)	
C5	0.6128 (3)	0.16289 (14)	−0.0011 (2)	0.0190 (6)	
H5A	0.6628	0.1799	−0.0486	0.023*	
C6	0.5978 (3)	0.19165 (14)	0.0902 (2)	0.0189 (6)	
C7	0.6566 (3)	0.24952 (15)	0.1137 (2)	0.0198 (6)	
C8	0.7805 (3)	0.24912 (17)	0.1459 (2)	0.0284 (8)	
C9	0.8530 (3)	0.2188 (2)	0.0673 (4)	0.0504 (13)	
H9A	0.8475	0.2409	0.0045	0.076*	
H9B	0.8263	0.1778	0.0572	0.076*	
H9C	0.9319	0.2181	0.0895	0.076*	
C10	0.7884 (4)	0.2155 (2)	0.2453 (4)	0.0553 (13)	
H10A	0.7413	0.2355	0.2951	0.083*	
H10B	0.8671	0.2150	0.2680	0.083*	
H10C	0.7619	0.1744	0.2362	0.083*	
C11	0.8247 (4)	0.3122 (2)	0.1619 (3)	0.0431 (10)	
H11A	0.8219	0.3342	0.0989	0.065*	
H11B	0.9029	0.3104	0.1857	0.065*	
H11C	0.7777	0.3326	0.2115	0.065*	
C12	0.5736 (3)	0.07978 (16)	−0.1236 (2)	0.0270 (7)	
H12A	0.6273	0.1027	−0.1639	0.041*	
H12B	0.5010	0.0773	−0.1585	0.041*	
H12C	0.6033	0.0394	−0.1127	0.041*	
C13	0.6566 (4)	0.4554 (2)	−0.0192 (3)	0.0449 (11)	
H13A	0.7272	0.4705	−0.0498	0.054*	
H13B	0.5984	0.4870	−0.0253	0.054*	
C14	0.6196 (5)	0.4029 (2)	−0.0716 (3)	0.0563 (13)	
H14A	0.6078	0.4127	−0.1420	0.084*	
H14B	0.5486	0.3886	−0.0426	0.084*	
H14C	0.6774	0.3717	−0.0660	0.084*	
C15	0.7467 (5)	0.4866 (3)	0.1318 (4)	0.0477 (14)*	0.812 (8)
H15A	0.7758	0.4693	0.1947	0.057*	0.812 (8)
H15B	0.8122	0.4966	0.0891	0.057*	0.812 (8)
C16	0.6824 (7)	0.5401 (4)	0.1531 (6)	0.081 (2)*	0.812 (8)
H16A	0.7310	0.5693	0.1867	0.121*	0.812 (8)
H16B	0.6184	0.5301	0.1964	0.121*	0.812 (8)
H16C	0.6542	0.5572	0.0907	0.121*	0.812 (8)
C15'	0.6749 (17)	0.5051 (3)	0.1147 (12)	0.0477 (14)*	0.188 (8)
H15C	0.6705	0.5310	0.0551	0.057*	0.188 (8)
H15D	0.6053	0.5118	0.1541	0.057*	0.188 (8)
C16'	0.771 (2)	0.5238 (17)	0.174 (2)	0.081 (2)*	0.188 (8)
H16D	0.7621	0.5660	0.1918	0.121*	0.188 (8)
H16E	0.8404	0.5186	0.1352	0.121*	0.188 (8)
H16F	0.7747	0.4996	0.2346	0.121*	0.188 (8)

### (1) Di-µ-bromido-µ-{2-(2,2-dimethylpropanimidoyl)-N-[2-(2,2-dimethylpropanimidoyl)-4-methylphenyl]-4-methylaniline}-bis[(diethyl ether)lithium] Atomic displacement parameters (Å^2^)

**Table:**

	*U*^11^	*U*^22^	*U*^33^	*U*^12^	*U*^13^	*U*^23^
Br1	0.0324 (2)	0.0367 (2)	0.02499 (19)	−0.00523 (16)	−0.00689 (14)	0.00029 (15)
O1	0.075 (2)	0.0360 (16)	0.0331 (14)	−0.0292 (16)	−0.0057 (15)	0.0070 (12)
N1	0.059 (3)	0.018 (2)	0.025 (2)	0.000	0.023 (2)	0.000
Li1	0.039 (3)	0.024 (3)	0.027 (3)	−0.005 (3)	−0.001 (3)	−0.001 (2)
N2	0.0241 (16)	0.0225 (14)	0.0240 (14)	−0.0015 (12)	0.0002 (12)	−0.0004 (11)
C1	0.037 (2)	0.0225 (16)	0.0228 (16)	0.0012 (14)	0.0149 (15)	0.0016 (13)
C2	0.047 (2)	0.0276 (19)	0.033 (2)	−0.0091 (17)	0.0263 (17)	−0.0012 (15)
C3	0.040 (2)	0.0206 (16)	0.0347 (19)	−0.0075 (16)	0.0144 (17)	−0.0003 (15)
C4	0.0249 (17)	0.0229 (16)	0.0192 (15)	0.0017 (14)	0.0034 (13)	0.0011 (13)
C5	0.0180 (15)	0.0220 (15)	0.0169 (14)	0.0003 (13)	0.0029 (12)	0.0052 (12)
C6	0.0188 (16)	0.0184 (15)	0.0195 (14)	0.0012 (12)	0.0065 (12)	0.0027 (12)
C7	0.0238 (16)	0.0248 (16)	0.0109 (12)	0.0002 (14)	0.0053 (12)	−0.0001 (12)
C8	0.0256 (18)	0.0340 (19)	0.0256 (17)	0.0025 (15)	−0.0007 (14)	−0.0076 (15)
C9	0.0213 (19)	0.075 (3)	0.055 (3)	0.009 (2)	−0.0015 (18)	−0.036 (2)
C10	0.053 (3)	0.066 (3)	0.046 (3)	−0.011 (3)	−0.022 (2)	0.019 (2)
C11	0.027 (2)	0.041 (2)	0.061 (3)	−0.0044 (18)	−0.0027 (19)	−0.014 (2)
C12	0.0327 (19)	0.0269 (18)	0.0215 (16)	−0.0048 (15)	0.0036 (14)	−0.0025 (14)
C13	0.046 (2)	0.049 (3)	0.040 (2)	−0.001 (2)	0.0007 (19)	0.019 (2)
C14	0.079 (4)	0.062 (3)	0.028 (2)	0.000 (3)	0.002 (2)	0.003 (2)

### (1) Di-µ-bromido-µ-{2-(2,2-dimethylpropanimidoyl)-N-[2-(2,2-dimethylpropanimidoyl)-4-methylphenyl]-4-methylaniline}-bis[(diethyl ether)lithium] Geometric parameters (Å, º)

**Table:**

Br1—Li1	2.504 (6)	C9—H9B	0.9800
Br1—Li1^i^	2.531 (7)	C9—H9C	0.9800
O1—C13	1.400 (5)	C10—H10A	0.9800
O1—C15	1.450 (6)	C10—H10B	0.9800
O1—C15'	1.454 (8)	C10—H10C	0.9800
O1—Li1	1.961 (7)	C11—H11A	0.9800
N1—C1^i^	1.399 (4)	C11—H11B	0.9800
N1—C1	1.399 (4)	C11—H11C	0.9800
N1—H1	0.66 (5)	C12—H12A	0.9800
Li1—N2	2.065 (7)	C12—H12B	0.9800
Li1—Br1^i^	2.531 (7)	C12—H12C	0.9800
Li1—Li1^i^	3.211 (13)	C13—C14	1.438 (7)
N2—C7	1.277 (4)	C13—H13A	0.9900
N2—H2	0.89 (4)	C13—H13B	0.9900
C1—C2	1.397 (5)	C14—H14A	0.9800
C1—C6	1.404 (4)	C14—H14B	0.9800
C2—C3	1.375 (5)	C14—H14C	0.9800
C2—H2A	0.9500	C15—C16	1.449 (9)
C3—C4	1.390 (5)	C15—H15A	0.9900
C3—H3A	0.9500	C15—H15B	0.9900
C4—C5	1.387 (5)	C16—H16A	0.9800
C4—C12	1.506 (4)	C16—H16B	0.9800
C5—C6	1.395 (4)	C16—H16C	0.9800
C5—H5A	0.9500	C15'—C16'	1.448 (10)
C6—C7	1.504 (4)	C15'—H15C	0.9900
C7—C8	1.528 (5)	C15'—H15D	0.9900
C8—C9	1.520 (5)	C16'—H16D	0.9800
C8—C11	1.521 (6)	C16'—H16E	0.9800
C8—C10	1.535 (6)	C16'—H16F	0.9800
C9—H9A	0.9800		
			
Li1—Br1—Li1^i^	79.3 (2)	C8—C10—H10A	109.5
C13—O1—C15	114.3 (3)	C8—C10—H10B	109.5
C13—O1—C15'	96.2 (7)	H10A—C10—H10B	109.5
C13—O1—Li1	126.3 (3)	C8—C10—H10C	109.5
C15—O1—Li1	118.2 (3)	H10A—C10—H10C	109.5
C15'—O1—Li1	117.3 (5)	H10B—C10—H10C	109.5
C1^i^—N1—C1	124.3 (4)	C8—C11—H11A	109.5
C1^i^—N1—H1	117.8 (2)	C8—C11—H11B	109.5
C1—N1—H1	117.8 (2)	H11A—C11—H11B	109.5
O1—Li1—N2	114.2 (3)	C8—C11—H11C	109.5
O1—Li1—Br1	116.4 (3)	H11A—C11—H11C	109.5
N2—Li1—Br1	113.8 (3)	H11B—C11—H11C	109.5
O1—Li1—Br1^i^	114.2 (3)	C4—C12—H12A	109.5
N2—Li1—Br1^i^	95.3 (3)	C4—C12—H12B	109.5
Br1—Li1—Br1^i^	100.1 (2)	H12A—C12—H12B	109.5
O1—Li1—Li1^i^	138.3 (2)	C4—C12—H12C	109.5
N2—Li1—Li1^i^	106.2 (2)	H12A—C12—H12C	109.5
Br1—Li1—Li1^i^	50.74 (16)	H12B—C12—H12C	109.5
Br1^i^—Li1—Li1^i^	50.01 (19)	O1—C13—C14	111.3 (4)
C7—N2—Li1	144.9 (3)	O1—C13—H13A	109.4
C7—N2—H2	111 (2)	C14—C13—H13A	109.4
Li1—N2—H2	101 (2)	O1—C13—H13B	109.4
C2—C1—N1	120.6 (3)	C14—C13—H13B	109.4
C2—C1—C6	118.2 (3)	H13A—C13—H13B	108.0
N1—C1—C6	121.1 (3)	C13—C14—H14A	109.5
C3—C2—C1	121.2 (3)	C13—C14—H14B	109.5
C3—C2—H2A	119.4	H14A—C14—H14B	109.5
C1—C2—H2A	119.4	C13—C14—H14C	109.5
C2—C3—C4	121.3 (3)	H14A—C14—H14C	109.5
C2—C3—H3A	119.3	H14B—C14—H14C	109.5
C4—C3—H3A	119.3	C16—C15—O1	109.9 (5)
C5—C4—C3	117.7 (3)	C16—C15—H15A	109.7
C5—C4—C12	121.6 (3)	O1—C15—H15A	109.7
C3—C4—C12	120.7 (3)	C16—C15—H15B	109.7
C4—C5—C6	122.2 (3)	O1—C15—H15B	109.7
C4—C5—H5A	118.9	H15A—C15—H15B	108.2
C6—C5—H5A	118.9	C15—C16—H16A	109.5
C5—C6—C1	119.4 (3)	C15—C16—H16B	109.5
C5—C6—C7	121.6 (3)	H16A—C16—H16B	109.5
C1—C6—C7	119.0 (3)	C15—C16—H16C	109.5
N2—C7—C6	119.8 (3)	H16A—C16—H16C	109.5
N2—C7—C8	120.3 (3)	H16B—C16—H16C	109.5
C6—C7—C8	119.8 (3)	C16'—C15'—O1	116 (2)
C9—C8—C11	108.6 (3)	C16'—C15'—H15C	108.3
C9—C8—C7	110.3 (3)	O1—C15'—H15C	108.3
C11—C8—C7	111.4 (3)	C16'—C15'—H15D	108.3
C9—C8—C10	110.5 (4)	O1—C15'—H15D	108.3
C11—C8—C10	108.2 (3)	H15C—C15'—H15D	107.4
C7—C8—C10	107.9 (3)	C15'—C16'—H16D	109.5
C8—C9—H9A	109.5	C15'—C16'—H16E	109.5
C8—C9—H9B	109.5	H16D—C16'—H16E	109.5
H9A—C9—H9B	109.5	C15'—C16'—H16F	109.5
C8—C9—H9C	109.5	H16D—C16'—H16F	109.5
H9A—C9—H9C	109.5	H16E—C16'—H16F	109.5
H9B—C9—H9C	109.5		

Symmetry code: (i) −*x*+1, *y*, −*z*+1/2.

### (2) Di-µ-bromido-2:3κ4Br:Br-bis{2-(2,2-dimethylpropanimidoyl)-N-[2-(2,2-dimethylpropanimidoyl)-4-methylphenyl]-4-methylaniline}-1κ3N,N',N'';4κ3N,N',N''-tetra-µ-isopropanolato-1:2κ4O:O;3:4κ4O:O-diisopropanolato-1κO,4κO-2,3-dilithium-1,4-dititanium Crystal data

**Table:**

[Li_2_Ti_2_Br_2_(C_24_H_32_N_3_)_2_(C_3_H_7_O)_6_]·1.5C_7_H_8_	*Z* = 1
*M**_r_* = 1761.71	*F*(000) = 860
Triclinic, *P*1	*D*_x_ = 1.309 Mg m^−^^3^
*a* = 12.2546 (3) Å	Cu *K*α radiation, λ = 1.54178 Å
*b* = 12.7240 (3) Å	Cell parameters from 16469 reflections
*c* = 15.9477 (5) Å	θ = 2.0–28.0°
α = 75.8613 (15)°	µ = 3.00 mm^−^^1^
β = 68.0449 (15)°	*T* = 100 K
γ = 83.2200 (17)°	Plates, orange
*V* = 2235.49 (11) Å^3^	0.25 × 0.20 × 0.04 mm

### (2) Di-µ-bromido-2:3κ4Br:Br-bis{2-(2,2-dimethylpropanimidoyl)-N-[2-(2,2-dimethylpropanimidoyl)-4-methylphenyl]-4-methylaniline}-1κ3N,N',N'';4κ3N,N',N''-tetra-µ-isopropanolato-1:2κ4O:O;3:4κ4O:O-diisopropanolato-1κO,4κO-2,3-dilithium-1,4-dititanium Data collection

**Table:**

Bruker APEXII DUO diffractometer	6139 reflections with *I* > 2σ(*I*)
Radiation source: IµS microsource	*R*_int_ = 0.083
phi and ω scans	θ_max_ = 66.5°, θ_min_ = 3.1°
Absorption correction: analytical [based on measured indexed crystal faces (SHELXTL2014; Sheldrick, 2008)]	*h* = −14→14
*T*_min_ = 0.667, *T*_max_ = 0.890	*k* = −15→14
30419 measured reflections	*l* = −18→18
7594 independent reflections	

### (2) Di-µ-bromido-2:3κ4Br:Br-bis{2-(2,2-dimethylpropanimidoyl)-N-[2-(2,2-dimethylpropanimidoyl)-4-methylphenyl]-4-methylaniline}-1κ3N,N',N'';4κ3N,N',N''-tetra-µ-isopropanolato-1:2κ4O:O;3:4κ4O:O-diisopropanolato-1κO,4κO-2,3-dilithium-1,4-dititanium Refinement

**Table:**

Refinement on *F*^2^	Primary atom site location: structure-invariant direct methods
Least-squares matrix: full	Secondary atom site location: difference Fourier map
*R*[*F*^2^ > 2σ(*F*^2^)] = 0.058	Hydrogen site location: mixed
*wR*(*F*^2^) = 0.172	H atoms treated by a mixture of independent and constrained refinement
*S* = 1.10	*w* = 1/[σ^2^(*F*_o_^2^) + (0.1092*P*)^2^ + 0.3301*P*] where *P* = (*F*_o_^2^ + 2*F*_c_^2^)/3
7594 reflections	(Δ/σ)_max_ = 0.005
386 parameters	Δρ_max_ = 0.72 e Å^−^^3^
324 restraints	Δρ_min_ = −0.43 e Å^−^^3^

### (2) Di-µ-bromido-2:3κ4Br:Br-bis{2-(2,2-dimethylpropanimidoyl)-N-[2-(2,2-dimethylpropanimidoyl)-4-methylphenyl]-4-methylaniline}-1κ3N,N',N'';4κ3N,N',N''-tetra-µ-isopropanolato-1:2κ4O:O;3:4κ4O:O-diisopropanolato-1κO,4κO-2,3-dilithium-1,4-dititanium Special details

**Table:**

Geometry. All esds (except the esd in the dihedral angle between two l.s. planes) are estimated using the full covariance matrix. The cell esds are taken into account individually in the estimation of esds in distances, angles and torsion angles; correlations between esds in cell parameters are only used when they are defined by crystal symmetry. An approximate (isotropic) treatment of cell esds is used for estimating esds involving l.s. planes.
Refinement. All H atoms were positioned geometrically ( C—H = 0.93/1.00 Å) and allowed to ride with *U*_iso_(H)= 1.2/1.5*U*_eq_(C). Methyl ones were allowed to rotate around the corresponding C—C.The asymmetric unit consists of the Ti dimer and one and a half toluene solvent molecules. The half toluene molecule is disordered around inversion symmetry while the one in general position is disordered in two parts. The toluene molecules were disordered and could not be modelled properly, thus program SQUEEZE, a part of the PLATON package of crystallographic software, was used to calculate the solvent disorder area and remove its contribution to the overall intensity data. The dimer also exhibits four disordered regions. The isopropyl groups on C25, C28, C31 and the t-butyl group on C21 are all disordered and each was refined in two parts. In each disordered case, the site occupation factors of the major and minor parts were fixed (only in the final cycle of refinement) to 0.6 and 0.4, respectively. The bridging Br ligand is also disordered and was refined in two parts, Br1 and Br2, with their site occupation factors refining to 0.68 (1) and 0.32 (1), respectively.

### (2) Di-µ-bromido-2:3κ4Br:Br-bis{2-(2,2-dimethylpropanimidoyl)-N-[2-(2,2-dimethylpropanimidoyl)-4-methylphenyl]-4-methylaniline}-1κ3N,N',N'';4κ3N,N',N''-tetra-µ-isopropanolato-1:2κ4O:O;3:4κ4O:O-diisopropanolato-1κO,4κO-2,3-dilithium-1,4-dititanium Fractional atomic coordinates and isotropic or equivalent isotropic displacement parameters (Å^2^)

**Table:**

	*x*	*y*	*z*	*U*_iso_*/*U*_eq_	Occ. (<1)
Ti1	−0.29130 (4)	0.27167 (4)	0.60831 (4)	0.03250 (17)	
Li1	−0.0775 (5)	0.3916 (4)	0.5292 (4)	0.0430 (11)	
Br1	0.13740 (19)	0.41263 (16)	0.4542 (4)	0.0495 (6)	0.674 (12)
Br2	0.1396 (4)	0.4153 (4)	0.4281 (4)	0.0495 (6)	0.326 (12)
O1	−0.4149 (2)	0.27962 (18)	0.57128 (16)	0.0442 (5)	
O2	−0.1909 (2)	0.34242 (17)	0.48909 (15)	0.0430 (5)	
O3	−0.15594 (17)	0.27994 (15)	0.64078 (14)	0.0342 (4)	
N1	−0.3643 (2)	0.39751 (19)	0.68255 (18)	0.0357 (5)	
H1	−0.323 (4)	0.448 (4)	0.664 (3)	0.048 (11)*	
N2	−0.3823 (2)	0.17604 (18)	0.73599 (17)	0.0343 (5)	
N3	−0.2387 (2)	0.1146 (2)	0.5799 (2)	0.0414 (6)	
H3	−0.230 (4)	0.116 (4)	0.518 (4)	0.077 (15)*	
C1	−0.4601 (3)	0.4005 (2)	0.7516 (2)	0.0386 (6)	
C2	−0.5434 (3)	0.3118 (2)	0.7792 (2)	0.0374 (6)	
C3	−0.5012 (3)	0.2035 (2)	0.7759 (2)	0.0366 (6)	
C4	−0.5854 (3)	0.1220 (3)	0.8087 (2)	0.0442 (7)	
H4A	−0.5594	0.0483	0.8112	0.053*	
C5	−0.7044 (3)	0.1470 (3)	0.8371 (3)	0.0511 (8)	
H5A	−0.7586	0.0902	0.8585	0.061*	
C6	−0.7471 (3)	0.2539 (3)	0.8352 (3)	0.0505 (8)	
C7	−0.6653 (3)	0.3340 (3)	0.8077 (2)	0.0423 (7)	
H7A	−0.6929	0.4069	0.8081	0.051*	
C8	−0.8773 (3)	0.2820 (3)	0.8636 (3)	0.0628 (11)	
H8A	−0.8902	0.3486	0.8216	0.094*	
H8B	−0.9075	0.2932	0.9271	0.094*	
H8C	−0.9187	0.2226	0.8604	0.094*	
C9	−0.4886 (3)	0.4914 (3)	0.8066 (2)	0.0448 (7)	
C10	−0.3744 (4)	0.5333 (3)	0.8038 (3)	0.0597 (10)	
H10A	−0.3935	0.5906	0.8389	0.089*	
H10B	−0.3252	0.5626	0.7393	0.089*	
H10C	−0.3316	0.4735	0.8312	0.089*	
C11	−0.5520 (5)	0.5852 (3)	0.7634 (3)	0.0677 (11)	
H11A	−0.5704	0.6424	0.7986	0.102*	
H11B	−0.6251	0.5602	0.7643	0.102*	
H11C	−0.5015	0.6140	0.6992	0.102*	
C12	−0.5613 (3)	0.4498 (3)	0.9095 (3)	0.0545 (8)	
H12A	−0.5698	0.5069	0.9433	0.082*	
H12B	−0.5211	0.3861	0.9350	0.082*	
H12C	−0.6393	0.4300	0.9158	0.082*	
C13	−0.2379 (3)	0.0185 (2)	0.6279 (2)	0.0453 (7)	
C14	−0.2662 (3)	0.0043 (2)	0.7277 (3)	0.0467 (8)	
C15	−0.3413 (3)	0.0789 (2)	0.7780 (2)	0.0409 (7)	
C16	−0.3732 (3)	0.0539 (3)	0.8745 (3)	0.0482 (8)	
H16A	−0.4255	0.1022	0.9092	0.058*	
C17	−0.3313 (3)	−0.0384 (3)	0.9212 (3)	0.0587 (9)	
H17A	−0.3539	−0.0519	0.9866	0.070*	
C18	−0.2549 (4)	−0.1125 (3)	0.8715 (3)	0.0649 (11)	
C19	−0.2254 (4)	−0.0897 (3)	0.7786 (3)	0.062 (1)	
H19A	−0.1742	−0.1396	0.7451	0.074*	
C20	−0.2074 (5)	−0.2153 (4)	0.9223 (4)	0.0935 (18)	
H20A	−0.1799	−0.2683	0.8828	0.140*	
H20B	−0.2703	−0.2465	0.9800	0.140*	
H20C	−0.1419	−0.1968	0.9366	0.140*	
C21	−0.2161 (5)	−0.0767 (5)	0.5729 (5)	0.0342 (16)*	0.6
C22	−0.2181 (5)	−0.0386 (6)	0.4752 (5)	0.0452 (15)*	0.6
H22A	−0.1568	0.0145	0.4387	0.068*	0.6
H22B	−0.2035	−0.1009	0.4459	0.068*	0.6
H22C	−0.2952	−0.0050	0.4780	0.068*	0.6
C23	−0.3127 (5)	−0.1588 (5)	0.6266 (5)	0.0413 (13)*	0.6
H23A	−0.3138	−0.1850	0.6900	0.062*	0.6
H23B	−0.3890	−0.1243	0.6281	0.062*	0.6
H23C	−0.2974	−0.2201	0.5961	0.062*	0.6
C24	−0.0911 (5)	−0.1281 (5)	0.5622 (5)	0.0447 (16)*	0.6
H24A	−0.0320	−0.0726	0.5274	0.067*	0.6
H24B	−0.0864	−0.1575	0.6236	0.067*	0.6
H24C	−0.0762	−0.1867	0.5288	0.067*	0.6
C21'	−0.2093 (8)	−0.0880 (8)	0.5985 (8)	0.039 (3)*	0.4
C22'	−0.2132 (10)	−0.0623 (10)	0.5063 (10)	0.057 (3)*	0.4
H22D	−0.1953	−0.1281	0.4813	0.085*	0.4
H22E	−0.2920	−0.0340	0.5087	0.085*	0.4
H22F	−0.1550	−0.0076	0.4663	0.085*	0.4
C23'	−0.3044 (8)	−0.1723 (8)	0.6603 (8)	0.046 (2)*	0.4
H23D	−0.3032	−0.1909	0.7233	0.070*	0.4
H23E	−0.3820	−0.1416	0.6616	0.070*	0.4
H23F	−0.2880	−0.2377	0.6350	0.070*	0.4
C24'	−0.0853 (8)	−0.1383 (7)	0.5917 (8)	0.041 (2)*	0.4
H24D	−0.0798	−0.1567	0.6534	0.061*	0.4
H24E	−0.0726	−0.2042	0.5673	0.061*	0.4
H24F	−0.0252	−0.0860	0.5502	0.061*	0.4
C25	−0.5266 (4)	0.2636 (4)	0.5714 (4)	0.0730 (12)	
H25A	−0.5860	0.2784	0.6308	0.088*	0.6
H25B	−0.5822	0.2328	0.6351	0.088*	0.4
C26	−0.5319 (8)	0.1375 (7)	0.5749 (6)	0.080 (2)*	0.6
H26A	−0.6087	0.1226	0.5751	0.119*	0.6
H26B	−0.4695	0.1184	0.5204	0.119*	0.6
H26C	−0.5208	0.0943	0.6312	0.119*	0.6
C27	−0.5598 (10)	0.3282 (9)	0.4990 (7)	0.094 (3)*	0.6
H27A	−0.6389	0.3094	0.5070	0.140*	0.6
H27B	−0.5598	0.4049	0.5002	0.140*	0.6
H27C	−0.5038	0.3153	0.4395	0.140*	0.6
C26'	−0.5114 (13)	0.1940 (12)	0.5077 (10)	0.081 (3)*	0.4
H26D	−0.5883	0.1817	0.5067	0.122*	0.4
H26E	−0.4605	0.2290	0.4454	0.122*	0.4
H26F	−0.4752	0.1244	0.5280	0.122*	0.4
C27'	−0.5690 (13)	0.3826 (11)	0.5299 (10)	0.080 (3)*	0.4
H27D	−0.6472	0.3790	0.5275	0.120*	0.4
H27E	−0.5726	0.4318	0.5696	0.120*	0.4
H27F	−0.5131	0.4095	0.4673	0.120*	0.4
C28	−0.2138 (4)	0.3758 (4)	0.4047 (3)	0.0673 (11)	
H28A	−0.2983	0.4006	0.4215	0.081*	0.6
H28B	−0.2992	0.3622	0.4231	0.081*	0.4
C29	−0.1426 (6)	0.4698 (6)	0.3430 (5)	0.0556 (15)*	0.6
H29A	−0.1599	0.4910	0.2858	0.083*	0.6
H29B	−0.1620	0.5306	0.3742	0.083*	0.6
H29C	−0.0588	0.4500	0.3280	0.083*	0.6
C30	−0.1992 (7)	0.2844 (6)	0.3591 (5)	0.0548 (16)*	0.6
H30A	−0.2155	0.3100	0.3016	0.082*	0.6
H30B	−0.1183	0.2549	0.3447	0.082*	0.6
H30C	−0.2541	0.2277	0.4006	0.082*	0.6
C29'	−0.2041 (18)	0.4866 (16)	0.3691 (13)	0.108 (5)*	0.4
H29D	−0.2522	0.5254	0.4174	0.163*	0.4
H29E	−0.1216	0.5061	0.3481	0.163*	0.4
H29F	−0.2315	0.5067	0.3168	0.163*	0.4
C30'	−0.153 (2)	0.297 (2)	0.3506 (18)	0.138 (9)*	0.4
H30D	−0.1722	0.2243	0.3888	0.206*	0.4
H30E	−0.1781	0.3076	0.2974	0.206*	0.4
H30F	−0.0682	0.3070	0.3287	0.206*	0.4
C31	−0.1278 (3)	0.2505 (3)	0.7214 (3)	0.0579 (9)	
H31A	−0.2022	0.2270	0.7744	0.069*	0.6
H31B	−0.1698	0.1822	0.7590	0.069*	0.4
C32	−0.0894 (5)	0.3535 (5)	0.7367 (4)	0.0479 (13)*	0.6
H32A	−0.0693	0.3352	0.7927	0.072*	0.6
H32B	−0.0206	0.3829	0.6832	0.072*	0.6
H32C	−0.1541	0.4079	0.7441	0.072*	0.6
C33	−0.0448 (6)	0.1628 (5)	0.7214 (5)	0.0565 (15)*	0.6
H33A	−0.0297	0.1471	0.7795	0.085*	0.6
H33B	−0.0761	0.0986	0.7157	0.085*	0.6
H33C	0.0289	0.1822	0.6691	0.085*	0.6
C32'	−0.1373 (16)	0.3123 (15)	0.7800 (12)	0.102 (5)*	0.4
H32D	−0.1114	0.2704	0.8299	0.153*	0.4
H32E	−0.0880	0.3755	0.7470	0.153*	0.4
H32F	−0.2196	0.3364	0.8063	0.153*	0.4
C33'	0.0222 (12)	0.2159 (12)	0.6785 (10)	0.082 (3)*	0.4
H33D	0.0365	0.1697	0.6342	0.123*	0.4
H33E	0.0674	0.2819	0.6474	0.123*	0.4
H33F	0.0468	0.1765	0.7298	0.123*	0.4

### (2) Di-µ-bromido-2:3κ4Br:Br-bis{2-(2,2-dimethylpropanimidoyl)-N-[2-(2,2-dimethylpropanimidoyl)-4-methylphenyl]-4-methylaniline}-1κ3N,N',N'';4κ3N,N',N''-tetra-µ-isopropanolato-1:2κ4O:O;3:4κ4O:O-diisopropanolato-1κO,4κO-2,3-dilithium-1,4-dititanium Atomic displacement parameters (Å^2^)

**Table:**

	*U*^11^	*U*^22^	*U*^33^	*U*^12^	*U*^13^	*U*^23^
Ti1	0.0346 (3)	0.0216 (3)	0.0388 (3)	−0.0095 (2)	−0.0098 (2)	−0.00359 (19)
Li1	0.037 (2)	0.029 (2)	0.055 (3)	−0.007 (2)	−0.006 (2)	−0.007 (2)
Br1	0.0345 (2)	0.0240 (2)	0.0692 (16)	−0.00711 (16)	0.0107 (7)	−0.0167 (6)
Br2	0.0345 (2)	0.0240 (2)	0.0692 (16)	−0.00711 (16)	0.0107 (7)	−0.0167 (6)
O1	0.0448 (11)	0.0411 (11)	0.0506 (13)	−0.0091 (10)	−0.021 (1)	−0.0069 (9)
O2	0.0516 (12)	0.0342 (11)	0.0392 (11)	−0.0132 (10)	−0.0142 (9)	0.0018 (8)
O3	0.0362 (10)	0.0279 (9)	0.0375 (11)	−0.0094 (8)	−0.0087 (8)	−0.0086 (8)
N1	0.0343 (12)	0.0220 (11)	0.0448 (14)	−0.0098 (10)	−0.0079 (10)	−0.0023 (9)
N2	0.0338 (11)	0.0207 (10)	0.0434 (13)	−0.0067 (9)	−0.0096 (10)	−0.0024 (9)
N3	0.0393 (13)	0.0314 (12)	0.0506 (16)	−0.0099 (11)	−0.0088 (11)	−0.0114 (11)
C1	0.0405 (15)	0.0246 (13)	0.0480 (17)	−0.0044 (12)	−0.0152 (13)	−0.0025 (11)
C2	0.0337 (14)	0.0293 (14)	0.0447 (17)	−0.0089 (11)	−0.0116 (12)	0.0001 (11)
C3	0.0372 (14)	0.0274 (13)	0.0424 (16)	−0.0060 (12)	−0.0146 (12)	0.0004 (11)
C4	0.0380 (15)	0.0301 (15)	0.057 (2)	−0.0088 (13)	−0.0144 (13)	0.0033 (13)
C5	0.0410 (16)	0.0331 (15)	0.071 (2)	−0.0157 (13)	−0.0179 (15)	0.0068 (14)
C6	0.0363 (16)	0.0429 (17)	0.067 (2)	−0.0073 (14)	−0.0194 (15)	0.0023 (15)
C7	0.0401 (15)	0.0307 (14)	0.0508 (19)	−0.0024 (12)	−0.0154 (13)	−0.0002 (12)
C8	0.0398 (17)	0.050 (2)	0.092 (3)	−0.0052 (16)	−0.0266 (18)	0.0032 (19)
C9	0.0468 (16)	0.0322 (15)	0.0541 (19)	−0.0002 (13)	−0.0143 (14)	−0.0133 (13)
C10	0.058 (2)	0.052 (2)	0.069 (2)	−0.0137 (17)	−0.0106 (17)	−0.0277 (18)
C11	0.088 (3)	0.0331 (17)	0.083 (3)	0.0070 (19)	−0.032 (2)	−0.0171 (17)
C12	0.0490 (18)	0.055 (2)	0.060 (2)	−0.0027 (16)	−0.0139 (16)	−0.0229 (16)
C13	0.0368 (15)	0.0298 (14)	0.0609 (19)	−0.0103 (13)	−0.0053 (14)	−0.0099 (13)
C14	0.0394 (15)	0.0252 (14)	0.064 (2)	−0.0077 (13)	−0.0091 (14)	−0.0015 (13)
C15	0.0362 (14)	0.0275 (13)	0.0525 (18)	−0.0112 (12)	−0.0121 (13)	0.0017 (12)
C16	0.0413 (16)	0.0406 (16)	0.0558 (19)	−0.0089 (14)	−0.0157 (14)	0.0026 (13)
C17	0.0489 (19)	0.054 (2)	0.055 (2)	−0.0084 (16)	−0.0140 (16)	0.0157 (16)
C18	0.052 (2)	0.0435 (19)	0.076 (2)	0.0017 (17)	−0.0137 (18)	0.0132 (17)
C19	0.053 (2)	0.0329 (17)	0.078 (2)	0.0023 (16)	−0.0109 (17)	0.0074 (16)
C20	0.081 (3)	0.066 (3)	0.099 (4)	0.007 (3)	−0.023 (3)	0.025 (3)
C25	0.053 (2)	0.091 (3)	0.095 (3)	−0.001 (2)	−0.039 (2)	−0.035 (3)
C28	0.080 (3)	0.065 (2)	0.051 (2)	−0.018 (2)	−0.027 (2)	0.0107 (18)
C31	0.054 (2)	0.068 (2)	0.053 (2)	−0.0237 (19)	−0.0206 (16)	−0.0049 (17)

### (2) Di-µ-bromido-2:3κ4Br:Br-bis{2-(2,2-dimethylpropanimidoyl)-N-[2-(2,2-dimethylpropanimidoyl)-4-methylphenyl]-4-methylaniline}-1κ3N,N',N'';4κ3N,N',N''-tetra-µ-isopropanolato-1:2κ4O:O;3:4κ4O:O-diisopropanolato-1κO,4κO-2,3-dilithium-1,4-dititanium Geometric parameters (Å, º)

**Table:**

Ti1—O1	1.805 (2)	C22—H22C	0.9800
Ti1—O2	1.901 (2)	C23—H23A	0.9800
Ti1—O3	1.934 (2)	C23—H23B	0.9800
Ti1—N2	2.069 (2)	C23—H23C	0.9800
Ti1—N3	2.130 (3)	C24—H24A	0.9800
Ti1—N1	2.136 (3)	C24—H24B	0.9800
Ti1—Li1	2.885 (5)	C24—H24C	0.9800
Li1—O2	1.950 (6)	C21'—C22'	1.444 (16)
Li1—O3	1.979 (5)	C21'—C23'	1.549 (14)
Li1—Br1	2.468 (6)	C21'—C24'	1.553 (13)
Li1—Br2	2.551 (7)	C22'—H22D	0.9800
Li1—Br1^i^	2.561 (5)	C22'—H22E	0.9800
Li1—Br2^i^	2.658 (7)	C22'—H22F	0.9800
Li1—Li1^i^	3.276 (10)	C23'—H23D	0.9800
Br1—Li1^i^	2.561 (5)	C23'—H23E	0.9800
Br2—Li1^i^	2.658 (7)	C23'—H23F	0.9800
O1—C25	1.406 (5)	C24'—H24D	0.9800
O2—C28	1.430 (5)	C24'—H24E	0.9800
O3—C31	1.406 (4)	C24'—H24F	0.9800
N1—C1	1.279 (4)	C25—C27	1.408 (11)
N1—H1	0.79 (5)	C25—C26'	1.453 (14)
N2—C15	1.389 (4)	C25—C26	1.601 (10)
N2—C3	1.394 (4)	C25—C27'	1.607 (14)
N3—C13	1.276 (4)	C25—H25A	1.0000
N3—H3	0.94 (5)	C25—H25B	1.0000
C1—C2	1.484 (4)	C26—H26A	0.9800
C1—C9	1.550 (4)	C26—H26B	0.9800
C2—C7	1.404 (4)	C26—H26C	0.9800
C2—C3	1.420 (4)	C27—H27A	0.9800
C3—C4	1.414 (4)	C27—H27B	0.9800
C4—C5	1.379 (5)	C27—H27C	0.9800
C4—H4A	0.9500	C26'—H26D	0.9800
C5—C6	1.396 (5)	C26'—H26E	0.9800
C5—H5A	0.9500	C26'—H26F	0.9800
C6—C7	1.390 (5)	C27'—H27D	0.9800
C6—C8	1.511 (5)	C27'—H27E	0.9800
C7—H7A	0.9500	C27'—H27F	0.9800
C8—H8A	0.9800	C28—C29'	1.39 (2)
C8—H8B	0.9800	C28—C30'	1.43 (3)
C8—H8C	0.9800	C28—C30	1.477 (9)
C9—C11	1.511 (5)	C28—C29	1.480 (8)
C9—C12	1.536 (5)	C28—H28A	1.0000
C9—C10	1.536 (5)	C28—H28B	1.0000
C10—H10A	0.9800	C29—H29A	0.9800
C10—H10B	0.9800	C29—H29B	0.9800
C10—H10C	0.9800	C29—H29C	0.9800
C11—H11A	0.9800	C30—H30A	0.9800
C11—H11B	0.9800	C30—H30B	0.9800
C11—H11C	0.9800	C30—H30C	0.9800
C12—H12A	0.9800	C29'—H29D	0.9800
C12—H12B	0.9800	C29'—H29E	0.9800
C12—H12C	0.9800	C29'—H29F	0.9800
C13—C14	1.465 (5)	C30'—H30D	0.9800
C13—C21'	1.501 (10)	C30'—H30E	0.9800
C13—C21	1.604 (7)	C30'—H30F	0.9800
C14—C15	1.416 (5)	C31—C32'	1.326 (17)
C14—C19	1.424 (5)	C31—C33	1.419 (8)
C15—C16	1.402 (5)	C31—C32	1.545 (7)
C16—C17	1.383 (5)	C31—C33'	1.749 (14)
C16—H16A	0.9500	C31—H31A	1.0000
C17—C18	1.413 (6)	C31—H31B	1.0000
C17—H17A	0.9500	C32—H32A	0.9800
C18—C19	1.352 (6)	C32—H32B	0.9800
C18—C20	1.536 (5)	C32—H32C	0.9800
C19—H19A	0.9500	C33—H33A	0.9800
C20—H20A	0.9800	C33—H33B	0.9800
C20—H20B	0.9800	C33—H33C	0.9800
C20—H20C	0.9800	C32'—H32D	0.9800
C21—C23	1.524 (8)	C32'—H32E	0.9800
C21—C22	1.524 (9)	C32'—H32F	0.9800
C21—C24	1.558 (8)	C33'—H33D	0.9800
C22—H22A	0.9800	C33'—H33E	0.9800
C22—H22B	0.9800	C33'—H33F	0.9800
			
O1—Ti1—O2	92.8 (1)	C24—C21—C13	108.3 (5)
O1—Ti1—O3	173.58 (9)	C21—C22—H22A	109.5
O2—Ti1—O3	82.72 (9)	C21—C22—H22B	109.5
O1—Ti1—N2	91.72 (10)	H22A—C22—H22B	109.5
O2—Ti1—N2	171.56 (10)	C21—C22—H22C	109.5
O3—Ti1—N2	93.31 (9)	H22A—C22—H22C	109.5
O1—Ti1—N3	92.29 (11)	H22B—C22—H22C	109.5
O2—Ti1—N3	92.89 (10)	C21—C23—H23A	109.5
O3—Ti1—N3	92.52 (10)	C21—C23—H23B	109.5
N2—Ti1—N3	79.8 (1)	H23A—C23—H23B	109.5
O1—Ti1—N1	92.3 (1)	C21—C23—H23C	109.5
O2—Ti1—N1	105.35 (10)	H23A—C23—H23C	109.5
O3—Ti1—N1	84.51 (9)	H23B—C23—H23C	109.5
N2—Ti1—N1	81.58 (9)	C21—C24—H24A	109.5
N3—Ti1—N1	160.94 (11)	C21—C24—H24B	109.5
O1—Ti1—Li1	131.18 (14)	H24A—C24—H24B	109.5
O2—Ti1—Li1	42.14 (14)	C21—C24—H24C	109.5
O3—Ti1—Li1	43.13 (13)	H24A—C24—H24C	109.5
N2—Ti1—Li1	135.69 (14)	H24B—C24—H24C	109.5
N3—Ti1—Li1	105.04 (13)	C22'—C21'—C13	103.8 (8)
N1—Ti1—Li1	85.61 (13)	C22'—C21'—C23'	108.1 (9)
O2—Li1—O3	80.3 (2)	C13—C21'—C23'	111.5 (7)
O2—Li1—Br1	131.2 (3)	C22'—C21'—C24'	107.5 (9)
O3—Li1—Br1	125.0 (3)	C13—C21'—C24'	115.7 (8)
O2—Li1—Br2	122.9 (3)	C23'—C21'—C24'	109.7 (8)
O3—Li1—Br2	130.7 (3)	C21'—C22'—H22D	109.5
Br1—Li1—Br2	8.92 (14)	C21'—C22'—H22E	109.5
O2—Li1—Br1^i^	106.9 (3)	H22D—C22'—H22E	109.5
O3—Li1—Br1^i^	114.7 (3)	C21'—C22'—H22F	109.5
Br1—Li1—Br1^i^	98.70 (18)	H22D—C22'—H22F	109.5
Br2—Li1—Br1^i^	100.0 (2)	H22E—C22'—H22F	109.5
O2—Li1—Br2^i^	111.8 (3)	C21'—C23'—H23D	109.5
O3—Li1—Br2^i^	107.9 (3)	C21'—C23'—H23E	109.5
Br1—Li1—Br2^i^	99.5 (2)	H23D—C23'—H23E	109.5
Br2—Li1—Br2^i^	102.1 (2)	C21'—C23'—H23F	109.5
Br1^i^—Li1—Br2^i^	8.51 (13)	H23D—C23'—H23F	109.5
O2—Li1—Ti1	40.86 (11)	H23E—C23'—H23F	109.5
O3—Li1—Ti1	41.91 (11)	C21'—C24'—H24D	109.5
Br1—Li1—Ti1	154.6 (2)	C21'—C24'—H24E	109.5
Br2—Li1—Ti1	152.1 (2)	H24D—C24'—H24E	109.5
Br1^i^—Li1—Ti1	106.67 (18)	C21'—C24'—H24F	109.5
Br2^i^—Li1—Ti1	105.5 (2)	H24D—C24'—H24F	109.5
O2—Li1—Li1^i^	136.3 (3)	H24E—C24'—H24F	109.5
O3—Li1—Li1^i^	139.3 (4)	O1—C25—C27	117.4 (6)
Br1—Li1—Li1^i^	50.58 (15)	O1—C25—C26'	108.0 (7)
Br2—Li1—Li1^i^	52.49 (17)	O1—C25—C26	106.6 (4)
Br1^i^—Li1—Li1^i^	48.12 (14)	C27—C25—C26	110.8 (6)
Br2^i^—Li1—Li1^i^	49.59 (17)	O1—C25—C27'	103.9 (6)
Ti1—Li1—Li1^i^	154.8 (3)	C26'—C25—C27'	108.9 (8)
Li1—Br1—Li1^i^	81.30 (18)	O1—C25—H25A	107.2
Li1—Br2—Li1^i^	77.9 (2)	C27—C25—H25A	107.2
C25—O1—Ti1	161.1 (3)	C26—C25—H25A	107.2
C28—O2—Ti1	129.1 (2)	O1—C25—H25B	111.9
C28—O2—Li1	131.4 (3)	C26'—C25—H25B	111.9
Ti1—O2—Li1	97.00 (19)	C27'—C25—H25B	111.9
C31—O3—Ti1	136.7 (2)	C25—C26—H26A	109.5
C31—O3—Li1	125.9 (3)	C25—C26—H26B	109.5
Ti1—O3—Li1	94.95 (19)	H26A—C26—H26B	109.5
C1—N1—Ti1	129.6 (2)	C25—C26—H26C	109.5
C1—N1—H1	118 (3)	H26A—C26—H26C	109.5
Ti1—N1—H1	112 (3)	H26B—C26—H26C	109.5
C15—N2—C3	117.2 (2)	C25—C27—H27A	109.5
C15—N2—Ti1	125.9 (2)	C25—C27—H27B	109.5
C3—N2—Ti1	116.09 (18)	H27A—C27—H27B	109.5
C13—N3—Ti1	135.9 (3)	C25—C27—H27C	109.5
C13—N3—H3	113 (3)	H27A—C27—H27C	109.5
Ti1—N3—H3	109 (3)	H27B—C27—H27C	109.5
N1—C1—C2	117.6 (3)	C25—C26'—H26D	109.5
N1—C1—C9	121.9 (3)	C25—C26'—H26E	109.5
C2—C1—C9	120.5 (3)	H26D—C26'—H26E	109.5
C7—C2—C3	119.0 (3)	C25—C26'—H26F	109.5
C7—C2—C1	120.5 (3)	H26D—C26'—H26F	109.5
C3—C2—C1	120.6 (3)	H26E—C26'—H26F	109.5
N2—C3—C4	119.2 (3)	C25—C27'—H27D	109.5
N2—C3—C2	123.0 (3)	C25—C27'—H27E	109.5
C4—C3—C2	117.6 (3)	H27D—C27'—H27E	109.5
C5—C4—C3	121.5 (3)	C25—C27'—H27F	109.5
C5—C4—H4A	119.3	H27D—C27'—H27F	109.5
C3—C4—H4A	119.3	H27E—C27'—H27F	109.5
C4—C5—C6	121.5 (3)	C29'—C28—C30'	122.7 (14)
C4—C5—H5A	119.3	C29'—C28—O2	112.7 (9)
C6—C5—H5A	119.3	C30'—C28—O2	105.7 (11)
C7—C6—C5	117.4 (3)	O2—C28—C30	111.6 (4)
C7—C6—C8	120.9 (3)	O2—C28—C29	111.1 (4)
C5—C6—C8	121.7 (3)	C30—C28—C29	112.8 (5)
C6—C7—C2	122.8 (3)	O2—C28—H28A	107.0
C6—C7—H7A	118.6	C30—C28—H28A	107.0
C2—C7—H7A	118.6	C29—C28—H28A	107.0
C6—C8—H8A	109.5	C29'—C28—H28B	104.7
C6—C8—H8B	109.5	C30'—C28—H28B	104.7
H8A—C8—H8B	109.5	O2—C28—H28B	104.7
C6—C8—H8C	109.5	C28—C29—H29A	109.5
H8A—C8—H8C	109.5	C28—C29—H29B	109.5
H8B—C8—H8C	109.5	H29A—C29—H29B	109.5
C11—C9—C12	110.9 (3)	C28—C29—H29C	109.5
C11—C9—C10	108.1 (3)	H29A—C29—H29C	109.5
C12—C9—C10	106.1 (3)	H29B—C29—H29C	109.5
C11—C9—C1	109.6 (3)	C28—C30—H30A	109.5
C12—C9—C1	111.7 (3)	C28—C30—H30B	109.5
C10—C9—C1	110.2 (3)	H30A—C30—H30B	109.5
C9—C10—H10A	109.5	C28—C30—H30C	109.5
C9—C10—H10B	109.5	H30A—C30—H30C	109.5
H10A—C10—H10B	109.5	H30B—C30—H30C	109.5
C9—C10—H10C	109.5	C28—C29'—H29D	109.5
H10A—C10—H10C	109.5	C28—C29'—H29E	109.5
H10B—C10—H10C	109.5	H29D—C29'—H29E	109.5
C9—C11—H11A	109.5	C28—C29'—H29F	109.5
C9—C11—H11B	109.5	H29D—C29'—H29F	109.5
H11A—C11—H11B	109.5	H29E—C29'—H29F	109.5
C9—C11—H11C	109.5	C28—C30'—H30D	109.5
H11A—C11—H11C	109.5	C28—C30'—H30E	109.5
H11B—C11—H11C	109.5	H30D—C30'—H30E	109.5
C9—C12—H12A	109.5	C28—C30'—H30F	109.5
C9—C12—H12B	109.5	H30D—C30'—H30F	109.5
H12A—C12—H12B	109.5	H30E—C30'—H30F	109.5
C9—C12—H12C	109.5	C32'—C31—O3	127.3 (8)
H12A—C12—H12C	109.5	O3—C31—C33	114.2 (4)
H12B—C12—H12C	109.5	O3—C31—C32	107.8 (4)
N3—C13—C14	118.2 (3)	C33—C31—C32	113.1 (4)
N3—C13—C21'	130.1 (5)	C32'—C31—C33'	104.6 (10)
C14—C13—C21'	111.7 (5)	O3—C31—C33'	102.7 (5)
N3—C13—C21	115.5 (4)	O3—C31—H31A	107.1
C14—C13—C21	126.1 (4)	C33—C31—H31A	107.1
C15—C14—C19	117.6 (4)	C32—C31—H31A	107.1
C15—C14—C13	121.6 (3)	C32'—C31—H31B	106.9
C19—C14—C13	120.6 (3)	O3—C31—H31B	106.9
N2—C15—C16	118.9 (3)	C33'—C31—H31B	106.9
N2—C15—C14	123.1 (3)	C31—C32—H32A	109.5
C16—C15—C14	118.0 (3)	C31—C32—H32B	109.5
C17—C16—C15	122.5 (4)	H32A—C32—H32B	109.5
C17—C16—H16A	118.8	C31—C32—H32C	109.5
C15—C16—H16A	118.8	H32A—C32—H32C	109.5
C16—C17—C18	120.0 (4)	H32B—C32—H32C	109.5
C16—C17—H17A	120.0	C31—C33—H33A	109.5
C18—C17—H17A	120.0	C31—C33—H33B	109.5
C19—C18—C17	117.8 (3)	H33A—C33—H33B	109.5
C19—C18—C20	121.6 (4)	C31—C33—H33C	109.5
C17—C18—C20	120.6 (4)	H33A—C33—H33C	109.5
C18—C19—C14	124.1 (4)	H33B—C33—H33C	109.5
C18—C19—H19A	117.9	C31—C32'—H32D	109.5
C14—C19—H19A	117.9	C31—C32'—H32E	109.5
C18—C20—H20A	109.5	H32D—C32'—H32E	109.5
C18—C20—H20B	109.5	C31—C32'—H32F	109.5
H20A—C20—H20B	109.5	H32D—C32'—H32F	109.5
C18—C20—H20C	109.5	H32E—C32'—H32F	109.5
H20A—C20—H20C	109.5	C31—C33'—H33D	109.5
H20B—C20—H20C	109.5	C31—C33'—H33E	109.5
C23—C21—C22	107.4 (6)	H33D—C33'—H33E	109.5
C23—C21—C24	112.3 (5)	C31—C33'—H33F	109.5
C22—C21—C24	106.1 (5)	H33D—C33'—H33F	109.5
C23—C21—C13	108.6 (5)	H33E—C33'—H33F	109.5
C22—C21—C13	114.2 (5)		

Symmetry code: (i) −*x*, −*y*+1, −*z*+1.
