# Supplementary material for: Crystal structures of a novel NNN pincer ligand and its dinuclear titanium(IV) alkoxide pincer complex
Source: Acta Crystallogr E Crystallogr Commun. 2017 Jan 6;73(Pt 2):122–6. doi: 10.1107/S2056989016019964 (PMC5290548; doi:10.1107/S2056989016019964)
Supplement: Supplementary file 4 [file e-73-00122-sup4.pdf]

Supporting Information

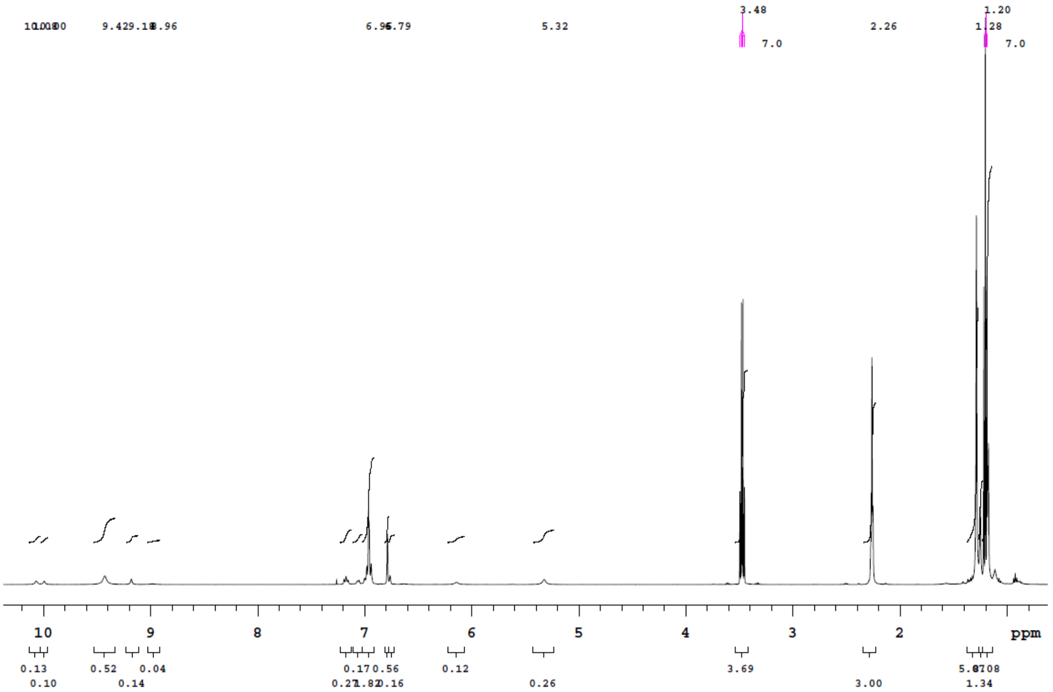

Figure S1. ^{1}H NMR – 500 MHz, $CDCl_3$ **[NNN]$H_3(LiBr)_2(Et_2O)_2$**

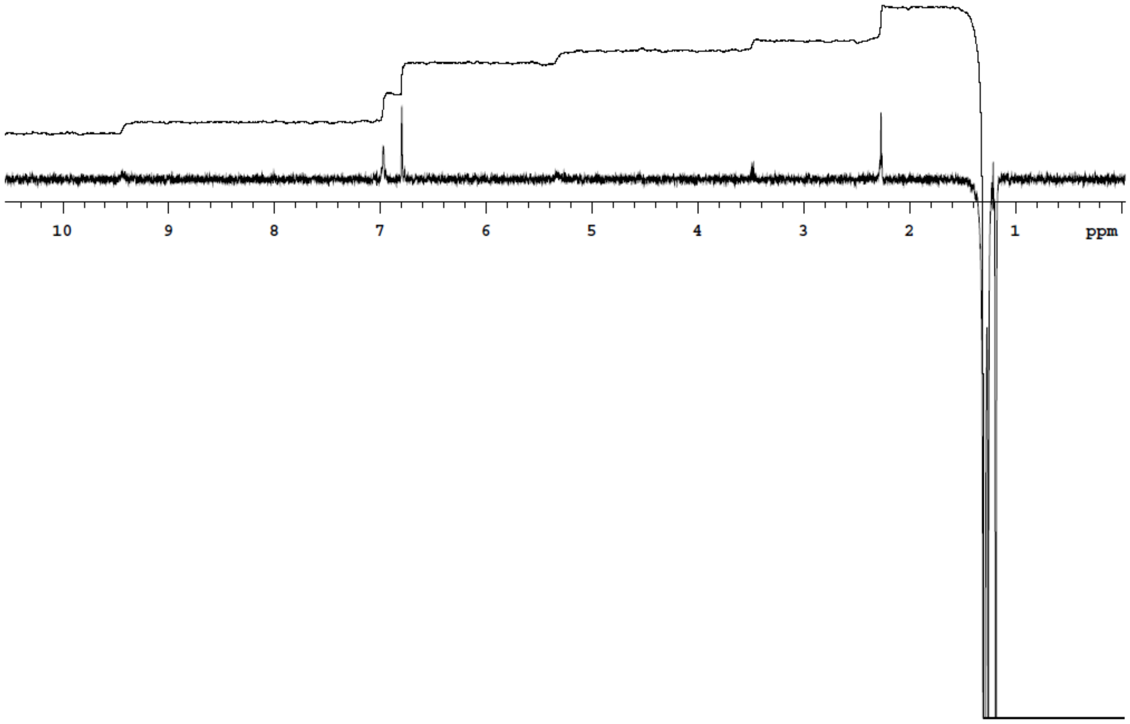

Figure S2. NOESY1D spectrum of **[NNN]$H_3(LiBr)_2(Et_2O)_2$** with selective irradiation at 1.28 ppm

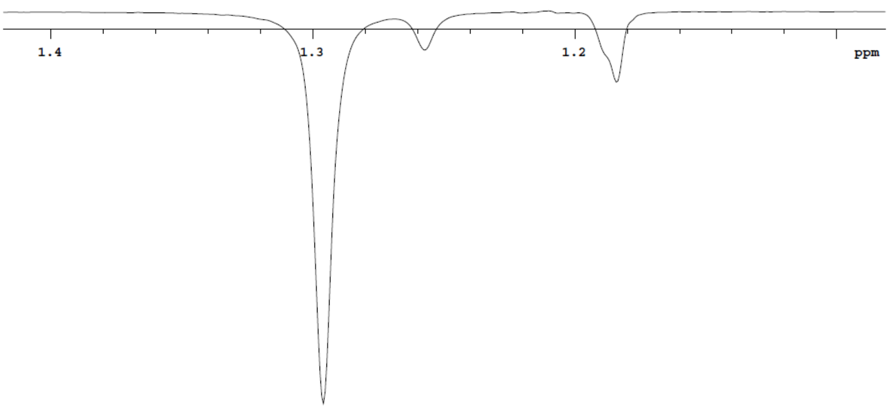

Figure S3. NOESY1D spectrum of **[NNN]$H_3(LiBr)_2(Et_2O)_2$** with selective irradiation at 1.28 ppm (expanded view)

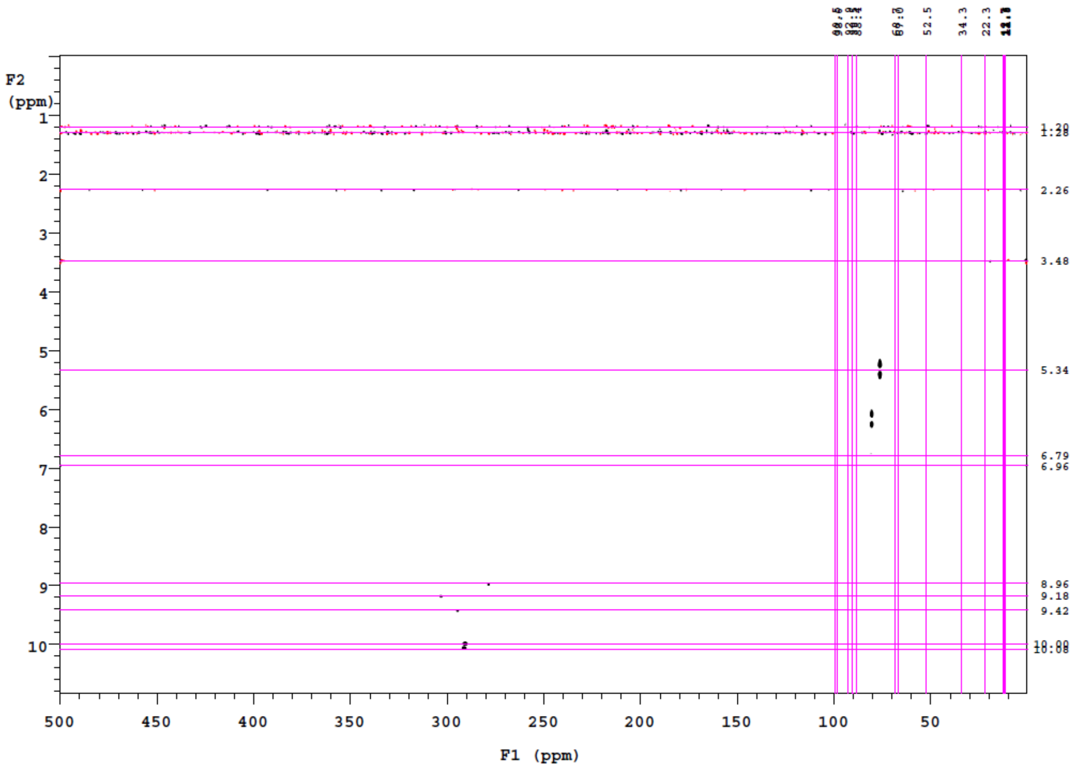

Figure S4. ^{1}H-^{13}C *g*HMBC ($CDCl_3$, 500 MHz, 25 °C) spectrum of complex **1.**

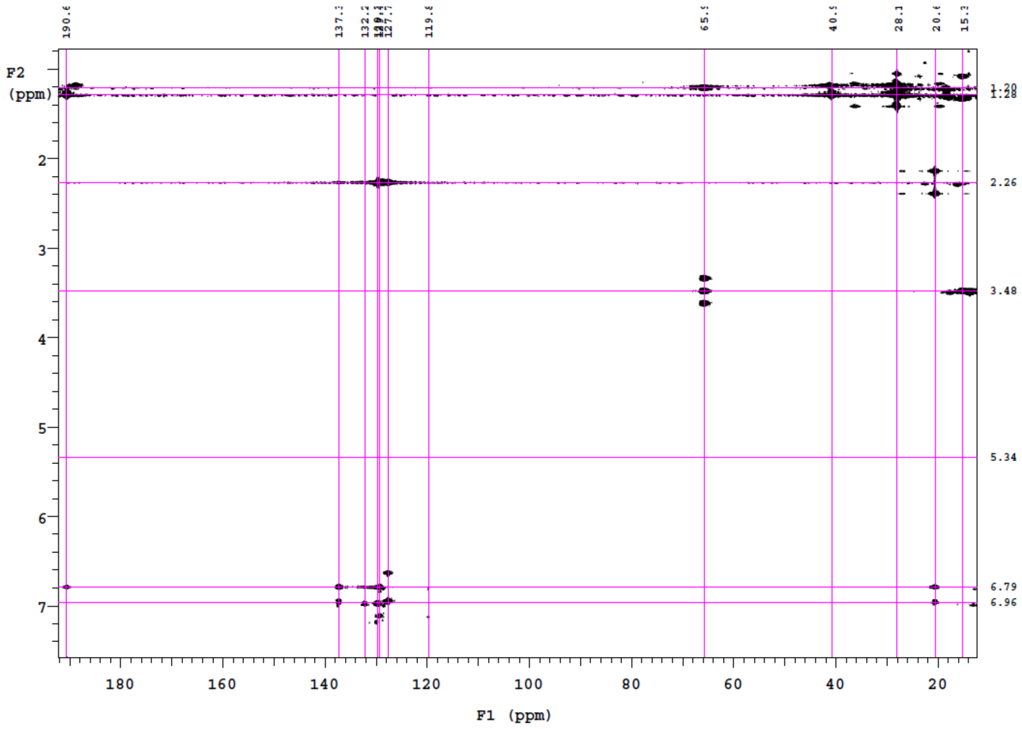

Figure S5. ^{1}H-^{13}C gHMBC ($CDCl_3$, 500 MHz, 25 °C) spectrum of complex **1,** expanded view

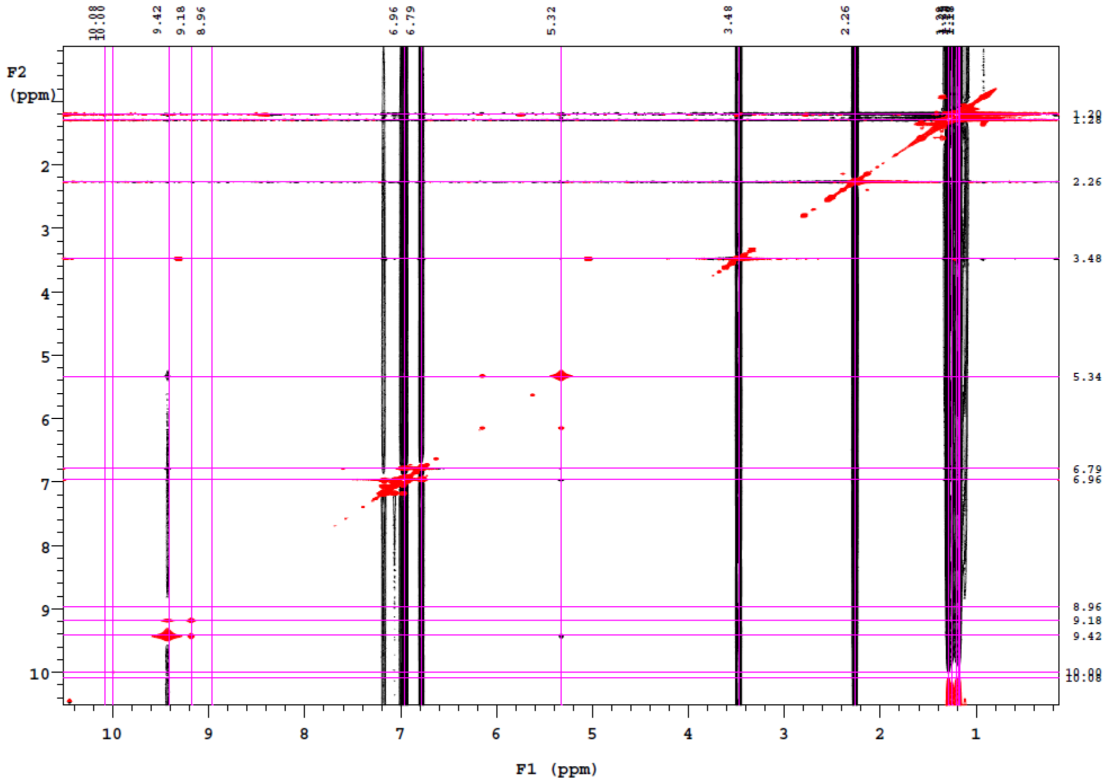

Figure S6. ^{1}H-^{13}C ROESY ($CDCl_3$, 500 MHz, 25 °C) spectrum of complex **1**

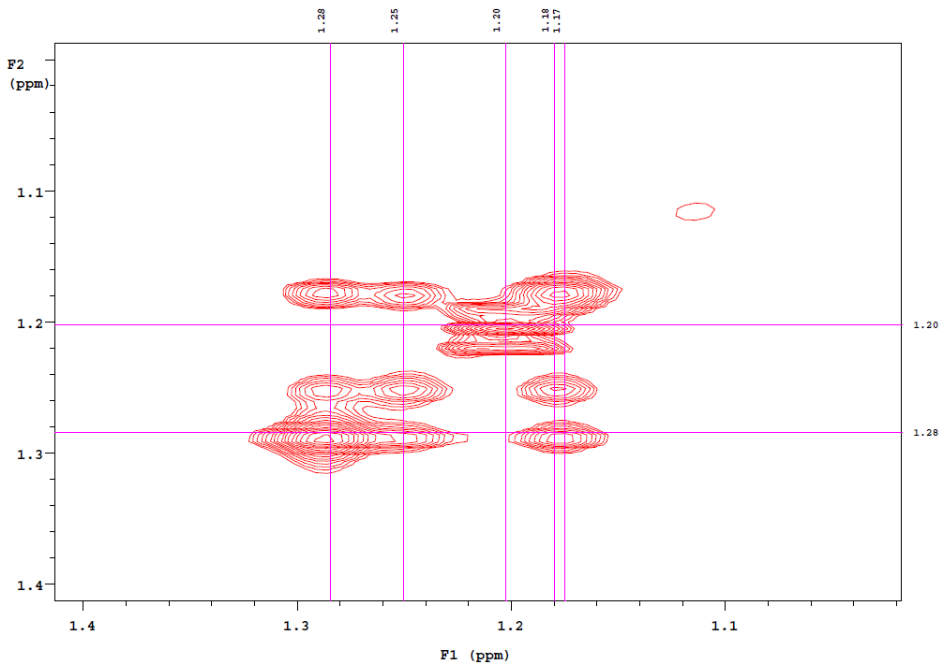

Figure S7. ^{1}H-^{1}H ROESY ($CDCl_3$, 500 MHz, 25 °C) spectrum of complex **1 ,** expanded view

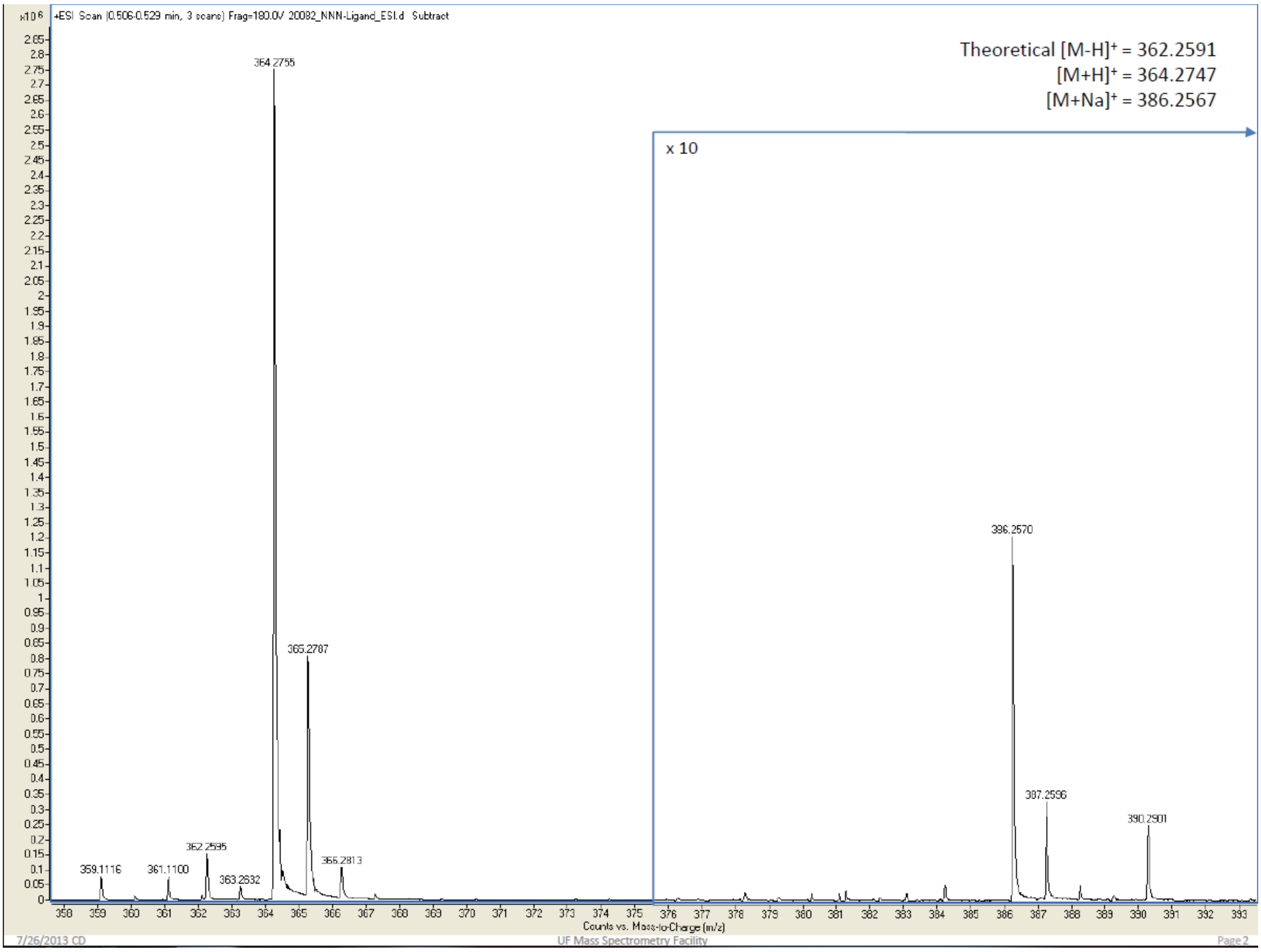

Figure S8 Mass spectrum of complex **1**
